# Risperidone plasma level, and its correlation with *CYP2D6* gene polymorphism, clinical response and side effects in chronic schizophrenia patients

**DOI:** 10.1186/s12888-023-05488-z

**Published:** 2024-01-10

**Authors:** Xiaoyi Wang, Jing Huang, Jianjun Lu, Xuemei Li, Hui Tang, Ping Shao

**Affiliations:** 1 Department of Psychiatry, National Clinical Research Center for Mental Disorders, and National Center for Mental Disorders, The Second Xiangya Hospital of Central South University, Changsha, 410011 Hunan China; 2 The Third People’s Hospital of Jiangyin City, Wuxi, Jiangsu China; 3 People’s Hospital of Dali Prefecture, Dali, Yunnan China

**Keywords:** Risperidone, Chronic schizophrenia, Plasma concentration, *CYP2D6* polymorphism, Effect, Safety

## Abstract

**Background:**

To explore the influence of *CYP2D6* genetic polymorphism on risperidone metabolism, thereby affecting risperidone’s effects and safeties in patients with chronic schizophrenia.

**Methods:**

Sixty-nine subjects with chronic schizophrenia treated with risperidone were recruited. *CYP2D6* genotypes was determined using targeted sequencing and translated into phenotype using activity system. Risperidone plasma concentrations were measured using HPLC. Positive and Negative Symptom Scale (PANSS) and Brief Psychiatric Rating Scale (BPRS) were used to evaluate the existence and severity of psychiatric symptoms, Barnes Akathisia Scale (BAS) and Extrapyramidal Symptom Rating Scale (ESRS) for neurological side effects. Metabolic and endocrine status assess were also included.

**Results:**

The plasma drug concentrations varied hugely among individuals. Intermediate metabolizer (IM) group had higher plasma levels of RIP and dose corrected RIP concentration, RIP/9-OH-RIP ratio and C/D ratio than normal metabolizer (NM) group (*p* < 0.01). There was no statistic difference between responders and non-responders in dose-adjusted plasma concentrations and ratios of RIP/9-OH-RIP and C/D. The occurrence of EPS was related to active moiety levels in 4th week (*p* < 0.05). The prolactin (PRL) levels in two follow-ups were both significantly higher than baseline (*p* < 0.01). PRL change from baseline to week 4 and week 8 were both positively associated with active moiety concentration detected in week 4 (*p* < 0.05).

**Conclusions:**

The risperidone plasma levels have great inter- and intraindividual variations, and are associated with the *CYP2D6* phenotypes, as well as the changes in serum prolactin in patients diagnosed with chronic schizophrenia.

**Supplementary Information:**

The online version contains supplementary material available at 10.1186/s12888-023-05488-z.

## Introduction

Risperidone (RIP), a first-line antipsychotic medication for schizophrenia, is a high-affinity antagonist of D_2_ and 5-HT_2A_, 5-HT_2C_ receptors. As an atypical antipsychotic or the second generation antipsychotic (SGA), risperidone has advantages in treating negative symptoms and cognition function, and causes less extrapyramidal symptoms (EPS) in comparing with typical antipsychotics [1]. However, those who take risperidone for treatment may have higher risks of suffering from glucose and lipid metabolic abnormalities and reproductive endocrine dysfunctions than those treated with FGAs. Risperidone is metabolized primarily by cytochrome P450 2D6 (CYP2D6) into the active metabolite 9-hydroxyrisperidone (9-OH-RIP), to a lesser extent by CYP3A4 and CYP3A5 [2]. This active metabolite is equivalent to risperidone for schizophrenia treatment, preclinical studies indicate that 9-hydroxy-risperidone has approximately 70% of the pharmacological activity of risperidone [3], so they are collectively called the “active moiety”.

Cytochrome P450 (P450) 2D6 is a major drug-metabolizing enzyme expressed in the liver. The *CYP2D6* gene is highly polymorphic, *CYP2D6* genetic variation impacts the metabolism of numerous drugs and, thus, can intensively impact drug efficacy and safety, and risperidone is one of them. To date, more than 160 allelic variants have been designated by the Pharmacogene Variation Consortium (PharmVar, https://www.pharmvar.org/gene/CYP2D6), most of them encode functional deficiency gene products and are distributed in different populations. Such as the decreased function CYP2D6*10 (100 C > T, P34S) allele has a relatively high frequency in Asian populations, including Chinese, Thai, Japanese, and Korean [4–9]. The *CYP2D6* allele variant with a high frequency among black Africans and African Americans is CYP2D6*17, but this allele is rarely observed in Europeans and their descendants [10, 11]. Conversely, the nonfunctional CYP2D6*4 allele is more frequent in European populations, CYP2D6*4 and CYP2D6*10 variants have been observed at very low to intermediate frequencies in African populations [12, 13]. Although the *CYP2D6* genetic polymorphism and its effect on the metabolizing activity of the CYP2D6 enzyme have been well-described, it is hard to translate the *CYP2D6* genotype into phenotype correctly. The traditional classification as poor (PM), intermediate (IM), extensive (EM, now referred to as normal (NM)), and ultrarapid (UM) metabolizer phenotype groups, or categorization by ‘‘number of active genes’’ often treating fully and reduced function alleles equally. To facilitate the translation process, a new rule-based system called Activity Score (AS) system, in which a genotype can be converted into an AS and then translated into phenotype, was first published in 2008 by Gaedigk et al. and widely adopted in the field including by Clinical Pharmacogenetics Implementation Consortium (CPIC) [14–16]. A consensus recommendation was achieved regarding the standardization of AS system using in translating *CYP2D6* genotype to phenotype by CPIC and the Dutch Pharmacogenetics Working Group (DPWG), and recently revised [17, 18]. It is essential to translate genotypes to phenotypes by using standardized method in research and clinical practice, so we employed this consensus-achieved system to analysis the data collected from subjects diagnosed with chronic schizophrenia treated with risperidone.

Except the genetic influences play an important role in determining the variability of the pharmacokinetic parameters of RIP [19, 20]. Previous studies also show us that the plasma levels of RIP and 9-OH-RIP varied widely among patients receiving similar doses of RIP, and the intra- and interindividual variations of RIP and 9-OH-RIP plasma levels were found to be large, thus resulting in inconsistent conclusions on correlations between the plasma concentrations and clinical responses and side-effects [21–24]. Furthermore, only a small number of patients with schizophrenia can be completely cured after antipsychotics therapy, the majority will develop a long-term, chronic course of the disease. And those patients may suffer from recurrence or relapse during the long-term course, and the therapy option needs to be adjusted at the same time, therefore, it is particularly important to manage this group more effectively.

Accordingly, we conduct this study to explore the influence of *CYP2D6* polymorphism on risperidone metabolism, thereby affecting risperidone’s effects and safety on patients diagnosed with chronic schizophrenia.

## Methods

### Study sample

Patients with chronic schizophrenia recruited in this study came from inpatients in the Third People’s Hospital of Jiangyin City, Jiangsu Province from May 2018 to May 2019. The inclusion criteria were as follows: (1) age 18–60 years old; (2) diagnosed as chronic schizophrenia according to the International Classification of Diseases, the Tenth Edition (ICD-10); (3) in an acute psychosis status; (4) without any antipsychotic medication in past 3 months; (5) treated with risperidone monotherapy. The exclusion criteria was as follows (patients who had following characteristics would be excluded from the study): (1) had been treated with electroconvulsive therapy within the past 3 months; (2) used other antipsychotics as adjunctive-therapy or combined use of antidepressants or mood stabilizers for treatment needs; (3) diagnosed as diabetes mellitus or other metabolites dysfunction; (4) suffered from other severe organic or neurological illness; (5) any other situation not suitable for participation in this study.

This study was approved by the ethics committee of the Third People’s Hospital of Jiangyin City. Written informed consent was obtained from every subject participated in this study.

### Study protocol

A baseline evaluation would be conducted by a well-trained investigator when candidates agreed to take part in this study. Those assessments include demographic data collection, medical history acquiring, physical examinations (vital signs and electrocardiograms, etc.), clinical symptoms measurements, and clinical laboratory tests. And Positive and Negative Symptom Scale (PANSS) was used to assess symptoms of schizophrenia, the Brief Psychiatric Rating Scale (BPRS) for psychiatric symptoms, and the Clinical Global Impression (CGI) for the severity of the illness. Clinical laboratory assessments include the fasting blood level of glucose, triglyceride, cholesterol, low-density lipoprotein, high-density lipoprotein, prolactin, and other laboratory tests for liver and renal function evaluation. Participants received risperidone monotherapy at a low dose which gradually increased to the targeted dose (2-6 mg/d) within one week according to disease status. No other medication was given except for benzodiazepines, which do not affect CYP2D6 activity or conversion of risperidone to 9-OH-RIP.

After that, patients should finish 3 follow-up visits at week 2 (visit 1), week 4 (visit 2), and week 8 (visit 3), respectively. During those 3 visits, apart from clinical and laboratory measurements mentioned above, adverse events of the participants were evaluated by Barnes Akathisia Scale (BAS) and Extrapyramidal Symptom Rating Scale (ESRS), assay for plasma concentration of risperidone (RIP) and 9-hydroxy-risperidone (9-OH-RIP). The total active moiety was defined as RIP plasma level plus 9-OH-RIP.

### *CYP2D*6 genotype analysis and phenotype

DNA was isolated from peripheral blood samples of 72 subjects and we genotyped 15 single nucleotide variants representing the most common *CYP2D6* alleles (*2, *3, *4, *5, *6, *7, *8, *9, *10, *14, *17, *29, *35, *41, *65) to maximize phenotype prediction, as well as the presence of duplications. DNA samples were checked for both the quantity and the quality by measuring absorbance at 260 and 280 nm (NanoDrop 2000, Thermo Scientific). The ratio of OD260nm/280nm of isolated DNA more than 1.7 was used for genetic analysis. DNA fragmentation was checked by agarose gel electrophoresis. Finally, 69 subjects were successfully genotyped. All blood sample processing, DNA extraction, and genotyping were blindly conducted by specialized technicians. When no allele-defining sequence variation was identified, CYP2D6*1 was assigned as the wild-type allele. Genotype analysis was performed by polymerase chain reaction (PCR) and following targeted sequencing. And the 15-interrogated variants including 14 *CYP2D6* single nucleotide polymorphisms (SNPs): 2851 C > T, 2550delA, 1847G > A, 1708delT, 2936 A > C, 1759G > T, 2616delAAG, 100 C > T, 1759G > T, 1022 C > T, 3184G > A, 31G > A, 2989G > A, and 4181G > C, as well as gene deletion and duplication. In those variants and SNPs, a CYP2D6*10 that had acquired 2850T and was designated CYP2D6*65 [25]. All variants were in Hardy-Weinberg equilibrium, except CYP2D6*10 (Table S1).

According to the CPIC guideline, normal function alleles (CYP2D6*1, *2, and *35) were assigned a value of 1 to calculate the AS; decreased function alleles (CYP2D6*9, *14, *17, *29 and *41) received a value of 0.5 while CYP2D6*10 0.25; and no function alleles (CYP2D6*3, *4, *5, *6, *7, and *8) received 0. The CYP2D6 activity score for each patient was the sum of the values assigned to each allele. CYP2D6 activity score was then used to classify patients with schizophrenia as intermediate metabolizers (IMs, activity score = 0.5 or 0.75), normal metabolizers (NMs, activity score = 1.25, 1.5 or 2.0).

### Assay for risperidone and 9-hydroxy-risperidone

The blood samples for RIP and 9-OH-RIP plasma level testing were obtained in the morning when subjects were in fasting status at every follow-up visit alongside other laboratory blood sampling. Plasma was separated by centrifuge from the collected blood samples and stored at -20℃ until assayed. Finally, high-performance liquid chromatography (HPLC) was used to determine the RIP and 9-OH-RIP concentration. Besides, the risperidone/9-hydroxyrisperidone (RIP/9-OH-RIP) ratio (a measure of CYP2D6), and the total risperidone concentration-to-dose (C/D) ratio (a measure of risperidone clearance in oral risperidone) were measured [26].

### Statistical analysis

All the data were analyzed using SPSS, version 25 (SPSS, Inc., Chicago).

The chi-square test was employed for the comparison of the counting variables, with Fisher’s exact tests when more than 20% of the variables had fewer than 5 subjects. Student’s t-test was employed for the quantitative variables, and Mann–Whitney non-parametric tests for non-normal distribution between two groups. Spearman’s correlation coefficient was calculated to examine correlations of variables with and without normal distribution. False Discovery Rate (FDR) was computed using the Benjamini-Hochberg method in multiple testing. Two-tailed *p* < 0.05 was considered statistically significant.

## Results

### Characteristics of participants

A total of 69 patients aged 18–60 years old diagnosed with chronic schizophrenia completed this study, and 33 of them were male. Table 1 manifested the baseline clinical and demographic characteristics of all participants.

**Table 1:** Clinical and demographic characteristics in follow-ups

Characteristics	Baseline	Week 4	Week 8
Sex Male/Female	33/36		
Age (years)	42.86 (11.60)		
Education (years)	9.25 (3.20)		
Illness duration (year)	14.01 (9.40)		
Daily oral dose (mg)	4.70 (1.15)		
PANSS	88.51 (7.89)	67.79 (10.66)	60.23 (11.39)
BPRS	46.19 (5.26)	33.91 (5.39)	19.98 (5.31)
CGI-S	5.17 (0.73)	3.71 (0.75)	2.95 (0.78)

All the data except sex was shown in mean (standard deviation) form

*PANSS* positive and negative symptoms scale, *BPRS* brief psychiatric rating scale, *CGI-S* clinical global impression-severe subscale

### Characteristics of drug plasma concentrations and their relationship with daily dose

**Fig. 1:**
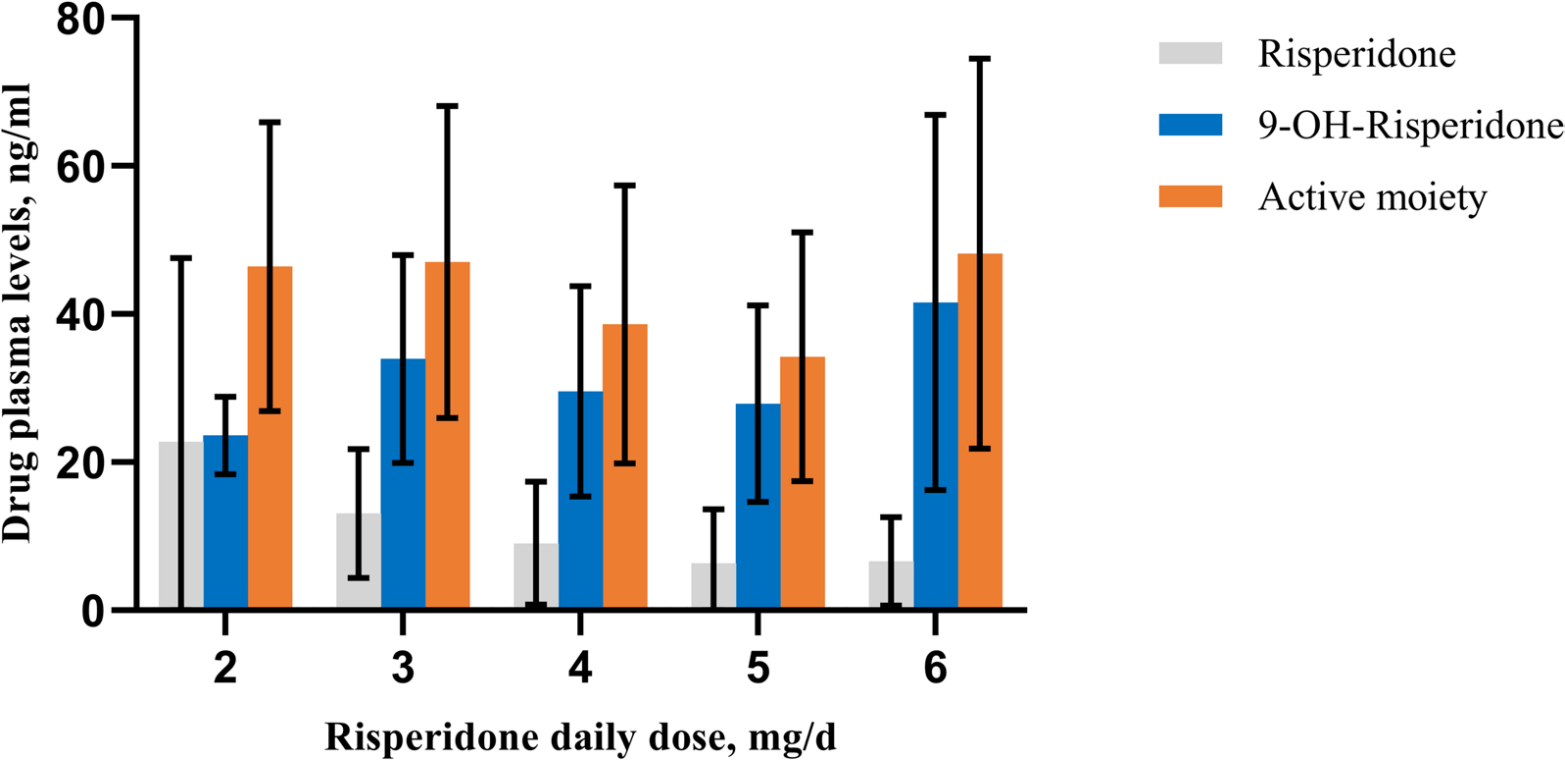
Drug plasma levels in every single dose level (2-6mg/d) in week 4. Data was shown in mean (standard deviation)

As the daily oral dose of those participants treated with risperidone reached to target level within 1 week and taking account of the time needed for steady-state plasma levels, the drug plasma level data used in this part come from 4-week visit. The mean drug plasma level in every single dose level (2–6 mg/d) in week 4 was described in Fig. 1. The plasma drug concentrations varied hugely among individuals and different doses of risperidone. The risperidone plasma level ranged from 1.9 ng/mL-40.5 ng/mL, 9-hydroxy-risperidone 1.9 ng/mL–82.4 ng/mL, and the active moiety 3.8 ng/mL–122.90 ng/mL. Risperidone plasma concentration had a significant negative but weaker correlation (*r*=-0.282, *p* = 0.030), while 9-hydroxy-risperidone had a significant positive relationship with risperidone daily dose (*r* = 0.410, *p* = 0.003). For the total active moiety and daily dose, there was no significant correlation (*r* = 0.234, *p* = 0.055). Figure S1 illustrated those mutual tendencies. Besides, no significant correlation was found between drug plasma levels and sex, age, weight and BMI, etc. Taking account of the recommended drug blood concentration range, 20-60ng/ml for active moiety, all the risperidone doses in this study could reach it. But these three daily doses accounted for the majority: 4 mg, 5 mg and 6 mg (*n* = 14, 11 and 13, respectively).

### Relationship between *CYP2D6* genotypes and phenotypes and plasma drug concentrations

In this part, we used dose-corrected plasma drug concentrations derived from visit 2 for analysis.

#### Distribution of the *CYP2D6* alleles and genotypes

Five *CYP2D6* alleles were detected in this study, including two normal function alleles and three decreased function alleles. Among them, the CYP2D6*10 decreased function allele was the most common allele identified among the 69 subjects at 59.42%. Another two decreased function alleles, CYP2D6*41 and CYP2D6*65, were observed at 1.45% and 6.52%, respectively. The frequencies of the normal function alleles CYP2D6*1 and CYP2D6*2 were 26.09% and 6.52%, respectively. *CYP2D6* allele frequencies were presented in Table 2. Of the 8 *CYP2D6* genotypes identified, CYP2D6*10/*10 was the most frequent (42.03%), followed by CYP2D6*1/*10, CYP2D6*10/*65, and CYP2D6*1/*1 (20.29%, 13.04%, and 11.59%, respectively). *CYP2D6* genotypes frequencies are presented in Table 3.

**Table 2:** *CYP2D6* allele frequencies (*n* = 69)

Alleles	CPIC clinical function	Frequency	%
*1	normal function	36	26.09%
*2	normal function	9	6.52%
*10	decreased function	82	59.42%
*41	decreased function	2	1.45%
*65	decreased function	9	6.52%

**Table 3:** *CYP2D6* genotype frequencies (*n* = 69), values assigned to each allele for activity score calculation, and the activity score of each genotype

Genotypes	Frequency (%)	Value allele 1	Value allele 2	Activity score
*10/*10	29 (42.03%)	0.25	0.25	0.5
*1/*10	14 (20.29%)	1	0.25	1.25
*10/*65	9 (13.04%)	0.25	0.5	0.75
*1/*1	8 (11.59%)	1	1	2
*1/*2	6 (8.70%)	1	1	2
*10/*41	1 (1.45%)	0.25	0.5	0.75
*2/*41	1 (1.45%)	1	0.5	1.5
*2/*2	1 (1.45%)	1	1	2

#### Plasma levels and C/D of risperidone, 9-OH-risperidone, active moiety and ratio in the different CYP2D6 phenotypes

Plasma levels of RIP and dose corrected RIP concentration in IM group were significantly higher than those in NM group (both *p*-value were < 0.001). Similarly, IMs had a significantly higher RIP/9-OH-RIP ratio and C/D ratio than those value in NMs (*p* = 0.009 and 0.003, respectively). However, we didn’t observe this kind of difference in blood levels of 9-OH-RIP and dose-corrected 9-OH-RIP, and active moiety (all *p* > 0.05). All those data were listed in Table 4.

**Table 4:** Mean dose-adjusted plasma concentrations and metabolic ratios in the different CYP2D6 phenotypes (*n* = 68)

	IMs (*n* = 38)	NMs (*n* = 30)	Statistics	*p*
RIP (ng/ml)	14.23 (10.79)	4.75 (3.93)	Z=-4.190	**< 0.001**
9-OH-RIP (ng/ml)	37.43 (18.82)	35.74 (14.55)	t = 407	0.685
Active moiety (ng/ml)	51.67 (24.62)	40.49 (13.76)	Z=-1.686	0.092
RIP/9-OH-RIP	0.44 (0.44)	0.20 (0.25)	t = 2.703	**0.009**
RIP/D (ng/ml/mg)	3.64 (3.45)	1.08 (1.09)	Z=-4.238	**< 0.001**
9-OH-RIP/D (ng/ml/mg)	8.89 (5.01)	7.20 (2.68)	Z=-1.204	0.092
C/D (ng/ml/mg)	12.53 (7.07)	8.28 (2.93)	Z=-2.940	**0.003**

Values expressed as mean (standard deviation). *RIP *Risperidone, *9-OH-RIP* 9-hydroxy-risperidone, Active moiety, the sum of risperidone plus 9-OH-RIP, *D* daily dose of oral risperidone, C/D, the total risperidone concentration-to-dose (C/D) ratio

Statistically significant *p* values are in bold

### Relationship between clinical outcomes and dose or drug plasma levels

For the outcomes assessed by PANSS, we defined those patients who had a percent ≥ 30% decrease from baseline to follow-up visits in PANSS total scores (subtracting 30 from the PANSS score so that a complete response is represented by 100% decrease) as responders, while < 30% as non-responders [23].

Comparisons were made between those two groups in aspects of age, sex, baseline symptoms severity, risperidone dose, and drug plasma levels in 2nd follow-up (4 week) visits, respectively. There were no significant differences between two groups in mean age and sex composition, so did in baseline symptoms severity valued by PANSS and BPRS (all *p* > 0.05). In 4-week visit, those patients in the response group took greater dose than in another group (*p*-value was < 0.001). Baseline illness severity in two groups was not different from each other. It obtained a significantly higher 9-OH-RIP concentration and active moiety level in responders in comparison to non-responders (*p* = 0.017, 0.046, respectively). The risperidone level in these two groups didn’t show a significant difference (*p* = 0.853). After adjusting the plasma drug concentrations by daily dose, there is no correlation existed, so does in RIP/9-OH-RIP ratio (all *p* > 0.05). All those data were listed in Table 5.

**Table 5:** Demographic and clinical characteristics, and plasma concentrations and metabolic ratios in study groups

	Responders (*n* = 39)	Non-responders (*n* = 29)	Statistics	*p*
Age	41.54 (11.60)	45.38 (10.86)	t = 1.387	0.170
Sex, M/F	17/22	15/14	χ^2^ = 0.442	0.625
Illness duration, year	12.59 (8.53)	16.21 (8.29)	t = 1.752	0.084
PANSS, baseline	89.13 (7.65)	87.69 (8.41)	t=-0.735	0.465
BPRS, baseline	46.08 (5.23)	46.45 (5.44)	t = 0.285	0.777
Dose	5.26 (0.99)	3.93 (0.92)	t=-5.609	**< 0.001**
RIP, PC	4.5 (2.4–14.20)	7.90 (2.65–15.90)	t=-0.187	0.853
9-OH-RIP, PC	41.00 (30.80–50.20)	27.80 (21.45–32.50)	t=-2.457	**0.017**
Active moiety	51.90 (35.70–63.40)	36.20 (30.35–43.85)	t=-2.032	**0.046**
RIP/9-OH-RIP	0.14 (0.07–0.39)	0.27 (0.12–0.49)	t = 1.016	0.313
RIP/D	0.93 (0.48-3.00)	1.98 (0.60–4.13)	t = 1.128	0.263
9-OH-RIP/D	8.00 (5.75–10.03)	6.88 (5.54–9.70)	t = 0.498	0.620
C/D	9.30 (6.68–9.30)	9.30 (7.03–15.87)	Z=-0.155	0.887

Data of age, illness duration, PANSS, BPRS, and dose were shown in mean (standard deviation) form. And the rest of the data except sex was presented as median (IQR)

Statistically significant *p* values are in bold

### Relationship between adverse events and dose or drug plasma levels

The common side effects of risperidone therapy mainly influence the extrapyramidal system, metabolic status and prolactin level. In this study, 25% of the subjects experienced extrapyramidal symptoms in visit 1, and 11.76% of them akathisia. And those two values increased to 32.79% and 13.11%, respectively (Table 6). As for extrapyramidal symptoms evaluated by using ESRS in last visit, whether or not its occurrence was related to total active moiety levels in 4th week (*r* = 0.428, *p* = 0.049), and those relations was not observed in 8th week (see in Supplementary materials). There was no correlation between extrapyramidal symptoms and 9-OH-RIP plasma concentration in both 4th week and 8th week. Whether in 4th or 8th week, there was no relationship existed between akathisia symptoms assessed by BAS and daily dose or drug plasma levels.

**Table 6:** Side effects of risperidone in follow-ups

Characteristics	Baseline	Week 4	Week 8	Differences (vs. Baseline)	
				Week 4	Week 8
ESRS, n (%)		17 (25%)	20 (32.79%)		
BAS, n (%)		8 (11.76%)	8 (13.11%)		
Weight (Kg)	66.25 (14.06)	64.99 (13.33)	64.79 (13.37)	-1.57 (2.96)^**^	-1.89 (3.42)^**^
BMI	24.51 (4.49)	24.04 (4.10)	24.07 (4.14)	-0.58 (1.11)^**^	-0.71 (1.27)^**^
Glu (mmol/L)	5.15 (0.86)	4.90 (1.20)	5.08 (1.25)	-0.35 (1.61)	-0.23 (1.28)
TG (mmol/L)	1.62 (1.09)	1.66 (0.89)	1.67 (0.94)	0.05 (0.96)	0.01 (1.11)
CHO (mmol/L)	4.77 (0.98)	4.50 (0.96)	4.54 (0.93)	-0.28 (0.78)^*^	-0.27 (0.84)^*^
LDL (mmol/L)	2.24 (0.64)	2.16 (0.65)	2.16 (0.65)	-0.09 (0.55)	-0.17 (0.74)
HDL (mmol/L)	1.17 (0.26)	1.10 (0.28)	1.10 (0.28)	-0.07 (0.23)^*^	-0.10 (0.33)^*^
PRL (ng/ml)	52.61 (41.35)	119.08 (59.49)	106.83 (60.80)	65.45 (63.01)^**^	52.40 (61.57)^**^

All the data except ESRS and BAS were shown in mean (standard deviation) form

*ESRS* Extrapyramidal Symptom Rating Scale, *BAS* Barnes Akathisia Scale, *BMI* body mass index, *Glu* fasting glucose, *TG* triglyceride, *CHO* cholesterol, *LDL* low-density lipoprotein, *HDL* high-density lipoprotein, *PRL* prolactin

^*^p < 0.05

^**^p < 0.01

In terms of the indexes reflecting metabolic status, 56.52% (*n* = 39) of patients with chronic schizophrenia were overweight at least (BMI ≥ 23 Kg/m^2^) in the baseline. During the following visits, however, weight, BMI, and fasting blood glucose and lipid levels (except triglyceride) were all shown a significant decreasing trend from baseline to two visits (all *p* < 0.05). We only observed an increasing trend in triglyceride, but this change was not significant (*p* > 0.05) (Table 6).

The prolactin (PRL) levels in week 4 and week 8 were both significantly higher than baseline (both *p* < 0.01, Table 6). Furthermore, we found PRL change from baseline to week 4 was positively associated with plasma concentration of active moiety in week 4 (*r* = 0.388, *p* = 0.049). Similarly, a significant correlation was also observed between PRL change from baseline to week 8 and active moiety level detected in week 4 (*r* = 0.388, *p* = 0.049). See in Supplementary materials.

## Discussions

In this study, we explored the correlation between dose and drug concentrations in chronic schizophrenia patients with oral risperidone treatment and observed the plasma drug concentrations varied hugely among individuals and different doses of risperidone. In addition, we observed that polymorphism of *CYP2D6* can significantly influence the metabolism of risperidone. As for the clinical response to risperidone therapy, the responders had higher 9-OH-RIP and active moiety levels than that of non-responders. Besides, drug-related EPS and the change in serum prolactin level were found to have a relationship with oral dose or plasma concentrations.

A few studies have suggested that there is a relationship existed between risperidone dose and plasma levels of risperidone and 9-hydroxy-risperidone. In exploring the inter- and intraindividual variations in RIP and 9-OH-RIP plasma levels, Aravagiri et al. found that there was a significantly strong relationship between the administered daily dose of RIP and plasma levels of 9-OH-RIP (*r* = 0.6654) and the active moiety (*r* = 0.7041), but not RIP plasma concentration, so it is important to measure steady-state levels of total active moiety by analyzing both RIP and 9-OH-RIP for plasma drug monitoring [22]. Riedel et al. in their research observed a positive but weak linear correlation between active moiety plasma level and dose (*r* = 0.291) [23]. Consistent with this finding, an association between RIP dose and 9-OH-RIP plasma concentration is also observed in our study, but this kind of correlation is not so strong. However, other researchers had different observations, a study focused on long-term treatment for chronic schizophrenia demonstrated that weight-normalized risperidone dosage had no correlation with plasma levels of risperidone, 9-hydroxy-risperidone or the active moiety [24]. There is still a consensus among these studies that serum concentrations of risperidone vary widely among individuals and different doses.

In this study, we compared the drug plasma levels at a steady state in relationship to *CYP2D6* genotypes. The metabolism of risperidone in vivo is mainly through 9-hydroxylation to produce 9-hydroxy-risperidone catalyzed by CYP2D6 in liver. Abundant researchers have established genetic polymorphisms of *CYP2D6* may play an important role in risperidone pharmacokinetics [20, 27], thereby affecting risperidone’s effects and safety. Among the genetic-polymorphism-related *CYP2D6* alleles, decreased function allele CYP2D6*10 has a relatively high frequency in Asians [4], and those who carry this allele may potentially have lower CYP2D6 activity than wild type [5, 28]. Accordingly, *CYP2D6* genotyping might be helpful for risperidone level assess, researchers found that *CYP2D6* poor metabolizers had greater dose-adjusted levels of risperidone and total active moiety, and higher RIP/9-OH-RIP ratio and lower dose-corrected 9-hydroxyrisperidone levels in first-episode drug-naïve schizophrenia patients, thereby indicating a lack of CYP2D6 activity [29]. Furthermore, two meta-analyses of pharmacogenetic studies on CYP2D6 also reported significantly higher drug exposure (dose-corrected plasma drug concentration or active moiety) in PM and IM phenotypes compared to the NM phenotype [30, 31]. The presence of the CYP2D6*10 allele was also associated with significantly higher dose-corrected risperidone levels and C/D ratio at week 12 in North Indian patients with schizophrenia, but they didn’t calculate the RIP/9-OH-RIP ratio [32]. In consistent with our results, a study in Thai ASD children and adolescents found that the plasma concentration of RIS, dose-corrected RIS, and RIS/9-OH-RIS ratio among IMs was significantly higher compared to that among NMs by using the revised CPIC method, however, they didn’t find a difference in C/D ratio which was inconsistent with our findings [33]. The same pattern was also observed in another study, Vanwong et al. also found that IMs had higher risperidone concentration and RIP/9-OH-RIP than NMs, moreover, the risperidone and RIP/9-OH-RIP ratio levels in the group with CYP2D6 AS 0.5 were significantly higher than the group with the CYP2D6 AS 2.0 [34, 35]. Taken together, RIP/9-OH-RIP ratio, an index of risperidone metabolize status, may be a stable biomarker for the CYP2D6 enzyme activity, no matter what kinds of patient risperidone treated with (including first episode or chronic schizophrenia, and ASD). In addition, dose-corrected risperidone and C/D ratio may play a similar role. Monitoring these indicators in clinical practice can help us manage the patients treated with antipsychotics more efficiently.

As for the relationships between drug plasma concentrations and clinical outcomes or adverse events are discrepancy. Some studies found that plasma concentration was associated with outcome, while others obtained opposite results [24, 36–38]. Previous studies failed to find an association between therapeutic effects and plasma drug concentrations [36, 39, 40]. In addition, Riedel, et al. suggested that plasma concentrations of the active moiety in responders were significantly lower than those in non-responders without significantly lower oral doses [23]. But Yasui-Furukori found that responders have higher plasma drug levels than that of non-responders [37]. In our study, daily dose and serum levels of 9-OH-RIP and active moiety were higher in response group than that in non-response group, while the ratios of risperidone/9-hydroxy-risperidone and C/D which were the indicative of risperidone metabolism and clearance didn’t show this kind of difference. Studies on risperidone in the treatment of children and adolescents with autism spectrum disorder (ASD), attention-deficit/hyperactivity disorder (ADHD), or other psychosis disorders have found that the sum trough concentrations of drug were significantly correlated with clinical outcomes and side effects [41, 42]. However, there is a lack of research evidence of adult groups concerning trough concentration. In a cohort recruited 150 psychiatric patients, a correlation between trough predicted concentration of the active moiety and neurologic symptoms (akathisia, tremor) was found, with no discuss on impact on clinical outcomes [20].

As for the drug-related side effects, it should be theoretically related to the drug dose. However, due to the large individual differences in plasma concentration and too many influencing factors, many studies on this aspect have not reached consistent conclusions. Nevertheless, high blood concentration (including risperidone and risperidone active metabolites) will still lead to increased risk of adverse drug reactions [20]. Therefore, based on these research results, the recommended total active moiety therapeutic range plasma concentration is 20–60 ng/ml, and laboratory alert plasma concentration is 150 mg/ml [43]. In the second generation of antipsychotics, a common adverse event is metabolic alterations, of which olanzapine has the highest incidence, which can develop to metabolic syndrome and cardiovascular disease [44–48]. This change emerges even after short exposure and increase with cumulative dosages. Although the incidence of risperidone is not so high, it is still reported by many studies [46, 49]. However, in our study, we did not find a significant relationship between plasma drug concentration and blood lipid levels, which may be related to chronic schizophrenia patients recruited in this study. At the same time, we even observed that weight and BMI, fasting glucose, and blood lipid levels were significantly decreased after a 4-week or 8-week of risperidone therapy. In several researches focused on antipsychotics treated chronic schizophrenia groups, researchers also found that patients may show a decrease or a mildly increase trend in metabolic status related indexes [50–52]. Data are controversial with regard to risperidone, and no study has as yet come to a definitive conclusion in assessing risperidone serum concentrations in association with metabolic outcomes [53]. Patients with chronic schizophrenia might have been treated with long-term or multiple antipsychotics in the past, and their metabolic characteristics may be different from those of the first-episode and drug naïve patients, which leads to a different change in metabolic related indicators when they are treated with risperidone again. Previous studies on risperidone in patients with chronic schizophrenia found the severity of extrapyramidal and anticholinergic symptoms (EPSE and ACS scores) tended to decrease during the study. In addition, there was no correlation between RIP or 9-OH-RIP plasma levels and EPSE or ACS scores, and no significant increase in mean body weight was recorded during the study [24]. Previous studies found the plasma threshold for parkinsonian side effects has been found to be 74 ng/mL, but they need to be confirmed [54, 55]. We also found a positive but not so strong correlation between active moiety concentration with occurring of EPS assessed by ESRS, it suggested patients may benefit from TDM as it can assist with monitoring of side effects. Unlike with other SGAs, risperidone therapy is often followed by a significant, dose-dependent elevation of prolactin levels. Both first episode and chronic patients experience significant prolactin increase [56, 57], and show a dose- and serum concentration-dependent elevation [58, 59]. In this study, we also found that PRL change from baseline to 4week was positively associated with plasma concentration of active moiety in 4week. Similarly, a significant correlation was also observed between PRL change from baseline to 8week and active moiety level detected in 4week, which means that AM concentration may be used to predict the degree of serum prolactin elevation.

The main limitation of this study was the small number of subjects. Previous studies also had the same drawback, having investigated small samples. Because the small number of subjects makes it difficult to detect small differences, may result in Type I error. Further, replication studies with larger number of subjects are required to confirm our findings. In addition, the plasma drug concentrations have great inter- and intraindividual variations, and influenced by many factors, especially the activity of CYP2D6. Hence it needs a more greater study sample to further confirm the findings.

In conclusion, we explore the characteristics of risperidone serum concentration and its main influencing factors, the *CYP2D6* polymorphism, as well as the relationship between plasma drug concentration and efficacy and adverse reactions on chronic schizophrenia patients. The results suggest that the plasma concentrations of risperidone, 9-hydroxyrisperidone and active moiety have great inter- and intraindividual variations, and are associated with the *CYP2D6* polymorphism. Besides, it related to changes in serum prolactin in patients diagnosed with chronic schizophrenia. But it seems to have no correlation with clinical response. These findings should be replicated with a large sample of subjects.

### Supplementary Information

**Additional file 1: Table S1.** CYP2D6 Alleles and Hardy-Weinberg Equilibrium Analysis. **Figure S1.** Relationship between risperidone dose after a 4-week treatment and plasma concentrations of risperidone (A), 9-hydroxy-risperidone (9-OH-R) (B) and active moiety (C) in 76 schizophrenic patients. **Table S2.** Correlations between plasma drug concentration (risperidone, 9-hydroxyrisperidone and active moiety), and side effects.

## Data Availability

All data generated or analyzed during this study are included in this published article and its supplementary information files.

## Abbreviations

SGA: The second-generation antipsychotic
EPS: Extrapyramidal symptoms
CYP2D6: Cytochrome P450 2D6
RIP: Risperidone
9-OH-RIP: 9-hydroxy-risperidone
PM: Poor metabolizer
IM: Intermediate metabolizer
EM: Extensive metabolizer
NM: Normal metabolizer
UM: Ultrarapid metabolizer
AS: Activity score
CPIP: Clinical Pharmacogenetics Implementation Consortium
DPWG: Dutch Pharmacogenetics Working Group
ICD-10: The International Classification of Diseases, the Tenth Edition (ICD-10)
PANSS: Positive and Negative Symptom Scale
BPRS: Brief Psychiatric Rating Scale
CGI: Clinical Global Impression
BAS: Barnes Akathisia Scale
ESRS: Extrapyramidal Symptom Rating Scale
PCR: Polymerase chain reaction
HPLC: High-performance liquid chromatography
C/D: Concentration-to-dose
BMI: Body mass index
PRL: Prolactin
ASD: Autism spectrum disorder
ADHD: Attention-deficit/hyperactivity disorder
