# Supplementary material for: Risperidone plasma level, and its correlation with CYP2D6 gene polymorphism, clinical response and side effects in chronic schizophrenia patients
Source: BMC Psychiatry. 2024 Jan 10;24:41. doi: 10.1186/s12888-023-05488-z (PMC10782740; doi:10.1186/s12888-023-05488-z)
Supplement: Supplementary file 1 — Additional file 1: Table S1. CYP2D6 Alleles and Hardy-Weinberg Equilibrium Analysis. Figure S1. Relationship between risperidone dose after a 4-week treatment and plasma concentrations of risperidone (A), 9-hydroxy-risperidone (9-OH-R) (B) and active moiety (C) in 76 schizophrenic patients. Table S2. Correlations between plasma drug concentration (risperidone, 9-hydroxyrisperidone and active moiety), and side effects. [file 12888_2023_5488_MOESM1_ESM.docx]

Table S1 CYP2D6 Alleles and Hardy-Weinberg Equilibrium Analysis

| Allele Name | dbSNP ID | Genotype Number | | | O(HET) | E(HET) | χ^2^ for Hardy-Weinberg Equilibrium | *p* |
| --- | --- | --- | --- | --- | --- | --- | --- | --- |
|  |  | Homozygous | Heterozygous | Wild-Type |  |  |  |  |
| *CYP2D6*2* | rs16947 | 1 | 17 | 51 | 0.2464 | 0.2375 | 1.877 | 0.619 |
| *CYP2D6*3* | rs35742686 | 0 | 0 | 69 | 0 | 0 | 0.000 | 1 |
| *CYP2D6*4* | rs3892097 | 0 | 0 | 69 | 0 | 0 | 0.000 | 1 |
| *CYP2D6*5* | del | 0 | 0 | 69 | 0 | 0 | 0.000 | 1 |
| *CYP2D6*6* | rs5030655 | 0 | 0 | 69 | 0 | 0 | 0.000 | 1 |
| *CYP2D6*7* | rs5030867 | 0 | 0 | 69 | 0 | 0 | 0.000 | 1 |
| *CYP2D6*8* | rs5030865 | 0 | 0 | 69 | 0 | 0 | 0.000 | 1 |
| *CYP2D6*9* | rs5030656 | 0 | 0 | 69 | 0 | 0 | 0.000 | 1 |
| *CYP2D6*10* | rs1065852 | 30 | 26 | 13 | 0.3768 | 0.4696 | 39.809 | <0.001 |
| *CYP2D6*14* | rs5030865 | 0 | 0 | 69 | 0 | 0 | 0.000 | 1 |
| *CYP2D6*17* | rs28371706 | 0 | 0 | 69 | 0 | 0 | 0.000 | 1 |
| *CYP2D6*29* | rs59421388 | 0 | 0 | 69 | 0 | 0 | 0.000 | 1 |
| *CYP2D6*35* | rs769258 | 0 | 0 | 69 | 0 | 0 | 0.000 | 1 |
| *CYP2D6*41* | rs28371725 | 0 | 2 | 67 | 0.0289 | 0.0286 | 0.000 | 0.690 |
| *CYP2D6*65* | rs16947, rs1065852 |  |  |  |  |  |  |  |

CYP2D6, Cvtochrome P450 2D6; dbSNP, Database of Single Nucleotide Polymorphisms; ID, identification number; E(HT), expected percentage of heterozygous; O(HET), observed percentage of heterozygous.

Figure S1 Relationship between risperidone dose after a 4-week treatment and plasma concentrations of risperidone (A), 9-hydroxy-risperidone (9-OH-R) (B) and active moiety (C) in 76 schizophrenic patients.

Table S2 Correlations between plasma drug concentration (risperidone, 9-hydroxyrisperidone and active moiety), and side effects

|  | Dose | RSP | | 9-OH-RSP | | Active moiety | |  |  |  | |  | |  | |
| --- | --- | --- | --- | --- | --- | --- | --- | --- | --- | --- | --- | --- | --- | --- | --- |
|  |  | 4week | 8week | 4week | 8week | 4week | 8week |  |  |  |  |  |  |  |  |
| ESRS 4w | r = 0.140 | r = 0.122 |  | r = 0.139 |  | r = 0.193 |  |  |  |  |  |  |  |  |  |
| ESRS 8w | r = 0.344 | r = 0.272 | r = 0.274 | r = 0.368 | r = 0.235 | r = 0.428^*^ | r = 0.311 |  |  |  |  |  |  |  |  |
| BAS 4w | r = 0.126 | r = -0.025 |  | r = -0.043 |  | r = -0.063 |  |  |  |  |  |  |  |  |  |
| BAS 8w | r = 0.153 | r = 0.151 | r = 0.078 | r = 0.168 | r = -0.084 | r = 0.179 | r = -0.051 |  |  |  |  |  |  |  |  |
| Weight 0-4w | r = -0.182 | r = 0.388^*^ |  | r = -0.051 |  | r = 0.049 |  |  |  |  |  |  |  |  |  |
| Weight 0-8w | r = -0.252 | r = 0.303 | r = 0.365^*^ | r = -0.126 | r = -0.112 | r = -0.015 | r = 0.040 |  |  |  |  |  |  |  |  |
| BMI 0-4w | r = -0.150 | r = 0.362^*^ |  | r = -0.059 |  | r = 0.029 |  |  |  |  |  |  |  |  |  |
| BMI 0-8w | r = -0.241 | r = 0.299 | r = 0.365^*^ | r = -0.149 | r = -0.123 | r = -0.034 | r = 0.027 |  |  |  |  |  |  |  |  |
| Glu 0-4w | r = 0.130 | r = -0.150 |  | r = 0.177 |  | r = 0.072 |  |  |  |  |  |  |  |  |  |
| Glu 0-8w | r = 0.020 | r = -0.003 | r = -0.043 | r = -0.018 | r = -0.129 | r = -0.069 | r = -0.137 |  |  |  |  |  |  |  |  |
| TG 0-4w | r = 0.112 | r = 0.005 |  | r = 0.020 |  | r = 0.013 |  |  |  |  |  |  |  |  |  |
| TG 0-8w | r = 0.248 | r = -0.011 | r = -0.020 | r = 0.105 | r = 0.174 | r = 0.103 | r = 0.156 |  |  |  |  |  |  |  |  |
| CHO 0-4w | r = 0.056 | r = -0.051 |  | r = 0.117 |  | r = 0.052 |  |  |  |  |  |  |  |  |  |
| CHO 0-8w | r = -0.020 | r = 0.191 | r = 0.036 | r = 0.120 | r = -0.010 | r = 0.186 | r = 0.028 |  |  |  |  |  |  |  |  |
| LDL 0-4w | r = 0.074 | r = -0.191 |  | r = -0.092 |  | r = -0.152 |  |  |  |  |  |  |  |  |  |
| LDL 0-8w | r = -0.159 | r = -0.009 | r = -0.121 | r = -0.152 | r = -0.134 | r = -0.115 | r = -0.105 |  |  |  |  |  |  |  |  |
| HDL 0-4w | r = -0.080 | r = -0.049 |  | r = -0.223 |  | r = -0.212 |  |  |  |  |  |  |  |  |  |
| HDL 0-8w | r = -0.162 | r = 0.235 | r = 0.084 | r = -0.106 | r=-0.382^*^ | r = 0.010 | r = -0.273 |  |  |  |  |  |  |  |  |
| PRL 0-4w | r = 0.041 | r = 0.202 |  | r = 0.322 |  | r = 0.388^*^ |  |  |  |  |  |  |  |  |  |
| PRL 0-8w | r = 0.272 | r = 0.044 | r = 0.009 | r = 0.329 | r = 0.133 | r = 0.388^*^ | r =0.107 |  |  |  |  |  |  |  |  |

*^*^p* < 0.05, *^**^p* < 0.01.
